# Comprehensive Identification of *AREB* Gene Family in *Populus euphratica* Oliv. and Functional Analysis of *PeAREB04* in Drought Tolerance

**DOI:** 10.3390/ijms26020518

**Published:** 2025-01-09

**Authors:** Binglei Liu, Jianhao Sun, Chen Qiu, Xiaoli Han, Zhijun Li

**Affiliations:** 1 Xinjiang Production & State Key Laboratory Incubation Base for Conservation and Utilization of Bio-Resource in Tarim Basin, Alar 843300, China; xiaogangliu6988@163.com (B.L.); sunjianhaotea@163.com (J.S.); qiuchentea@163.com (C.Q.); lilyan0509@163.com (X.H.); 2 College of Life Science and Technology, Tarim University, Research Center of Populus euphratica, Alar 843300, China

**Keywords:** *P. euphratica*, drought stress, *AREB*, stomatal openings

## Abstract

The transcription factors in the ABA Response Element Binding (AREB) protein family were differentially regulated under multiple stress conditions; however, functional analyses of AREB in *Populus euphratica* Oliv. had not been conducted previously. In the present study, the comprehensive identification of the *P. euphratica AREB* gene family and the function of *PeAREB04* in response to drought stress in *P. euphratica* were elucidated. A comprehensive analysis of the *PeAREB* family was first performed, followed by the determination of their expression patterns under drought stress. Bioinformatics analysis revealed that thirteen *AREB* genes were identified across the *P. euphratica* genome, with these genes distributed across eight chromosomes in a seemingly random pattern. Phylogenetic analysis indicated that the *PeAREB* genes could be categorized into four distinct branches. Cis-acting element analysis revealed that most *PeAREB* genes contained multiple hormone- and stress-responsive elements. Transcriptomic sequencing of *P. euphratica* seedlings under drought stress showed that most *PeAREB* genes responded rapidly to drought stress in either the leaves or roots. One gene, *PeAREB04*, was selected for further functional validation due to its significant upregulation in both leaves and roots under drought stress. Overexpression of *PeAREB04* in *Arabidopsis thaliana* resulted in a high survival rate, reduced water loss in isolated leaves, and a significant reduction in stomatal aperture under natural drought conditions. Drought stress simulations using mannitol further demonstrated that overexpression of *PeAREB04* significantly enhanced root elongation. These findings indicate that the identification of the *PeAREB* gene family and the characterization of *PeAREB04*’s role in drought stress have been largely accomplished. Furthermore, the *PeAREB04* gene demonstrates considerable potential as a key target for future genetic engineering strategies aimed at enhancing plant drought resistance.

## 1. Introduction

*Populus euphratica* is not only a foundational species of desert oases but also a rare and relict plant found in extreme arid deserts [1,2]. As such, *P. euphratica* is ideal model for studying abiotic stress responses in desert woody plants. Although *P. euphratica* exhibits exceptional drought tolerance, drought remains a significant limiting factor for the growth of its seedlings and population succession. Low osmotic potential markedly inhibits the growth of *P. euphratica* seedlings [3]. The survival rate of *P. euphratica* seedlings decreases under drought stress, and embryonic root growth is inhibited [4,5]. Therefore, the identification and functional validation of drought-resistant genes in *P. euphratica* are crucial to understand the mechanisms by which *P. euphratica* seedlings respond to drought and enhance their survival rates in extreme desert environments.

Nine members of the *AREB* gene family have been identified in *Arabidopsis thaliana*: *AtABF1*, *AtABF2*/*AREB1*, *AtABF3*, *AtABF4*/*AREB2*, *AtABI5*/*DPBF1*, *AtDPBF2*, *AtDPBF3*/*AREB3*, *AtDPBF4* and *AtbZIP15*. These genes belong to a subfamily of the bZIP gene family [6]. AREB transcription factors are closely linked to abiotic stress responses; in particular, ABF2/AREB1 plays a crucial role in drought and ABA stress responses. Overexpression of *AREB1* in transgenic *A. thaliana* enhances drought tolerance compared with WT plants [7,8]. The expression of numerous drought stress-responsive genes is induced by ABA, and AREB transcription factors function as key regulatory molecules in the ABA-signalling pathway. Under conditions of water deficit, AREB transcription factors initiate the expression of downstream genes by binding to ABRE cis-acting elements, thereby activating ABA-regulated gene expression [9,10,11]. This process significantly increases endogenous ABA levels, which enhance drought tolerance by maintaining tissue water balance, regulating stomatal openings, promoting root permeability and improving water conductivity [12,13]. In the ABA-signalling pathway, ABA binds to pyraclostrobin resistance 1 (PYR1), resulting in the inactivation of protein phosphatase 2C (PP2C). This phenomenon disrupts the interaction between PP2C and Snf1-related protein kinase 2 (SnRK2), thereby stimulating SnRK2 activity to activate AREB. This activation ultimately leads to the expression of target genes responsive to ABA signalling, including LEA protein genes and associated regulatory genes [14,15,16]. Conserved ABRE cis-acting elements have been identified in the promoter regions of functional resistance genes, such as *ICK1*, *RD29B*, *RAB18*, *KIN2* and *SUS1* [17,18].

In addition to being widely studied in the model plant *A. thaliana*, the *AREB* gene has important functions in other species, such as sandalwood [19], tomato [20] and Rosaceae [21]. However, the *AREB* gene family in *P. euphratica* has not yet been characterised. The present study leverages three generations of *P. euphratica* genome sequencing to explore its *AREB* gene family comprehensively. Firstly, we analysed the *PeAREB* gene family and focused on gene chromosomal localisation, gene duplication patterns, cis-acting elements, conserved gene motifs, protein structural domains, collinearity and multi-species phylogenetic relationships. Secondly, transcriptome data were obtained from *P. euphratica* subjected to drought stress and used to analyse the expression patterns of *PeAREB* genes in roots and leaves under drought conditions. The subcellular localisation of PeAREB04 was determined to identify its site of action. We also performed heterologous expression of *PeAREB04* in *A. thaliana* to investigate its role in drought stress response in *P. euphratica* [22]. This study offers essential data to advance the functional characterisation of the *PeAREB* gene family and elucidate the specific role of *PeAREB04* in plant responses to drought stress.

## 2. Results

### 2.1. Identification and Characterisation of PeAREB Genes

Nine *Arabidopsis* AREB protein sequences were used as references. Thirteen *PeAREB* genes were identified in *P. euphratica* through homology matching. These genes were designated as *PeAREB01* to *PeAREB13* according to their chromosomal positions. Analysis of the physicochemical properties of the encoded proteins revealed that PeAREB proteins contain 266 to 455 amino acid residues, with molecular weights ranging from 29,801.56 to 49,487.98 kDa and isoelectric points between 4.94 and 10 (Table 1). PeAREB02, PeAREB06, PeAREB07, PeAREB12 and PeAREB13 were classified as acidic proteins (pI < 7), while the remaining proteins were basic (pI > 7). The protein instability index for all genes exceeded 40, suggesting potential instability, and the aliphatic amino acid index ranged from 62.46 to 72.9. The grand average of hydropathy (GRAVY) values for all proteins were negative, ranging from −0.879 to −0.586, with more negative values indicating higher hydrophilicity. These findings suggest that PeAREB proteins were hydrophilic.

**Figure 1:**
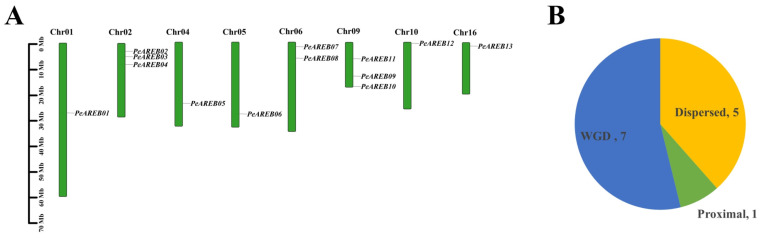
Chromosomal localization and gene family replication type of *PeAREB* genes. (**A**) Analysis of the chromosomal localisation of *PeAREBs*. (**B**) Analysis of *PeAREB* gene family replication types.

The *PeAREB* genes were unevenly distributed across the eight chromosomes of *P. euphratica*, with chromosome 2 (*PeAREB02*, *PeAREB03* and *PeAREB04*) and chromosome 9 (*PeAREB09*, *PeAREB10* and *PeAREB11*) containing the highest number of genes, each with three. By contrast, chromosome 1 (*PeAREB01*), chromosome 4 (*PeAREB05*), chromosome 5 (*PeAREB06*), chromosome 10 (*PeAREB12*) and chromosome 16 (*PeAREB13*) each contained only one gene (Figure 1A). Gene duplication events are crucial for the generation of new functions and genome evolution. An analysis of the gene family replication types of the 13 *PeAREB* genes revealed that whole-genome duplication (WGD) or segmental duplication was the most prevalent mechanism (seven instances), followed by dispersed duplication (five instances), while proximal duplication was the least common (one instance) (Figure 1B). These findings suggest that WGD or segmental duplication events were the primary drivers of *PeAREB* gene expansion, with varying evolutionary contributions from each chromosome to the *PeAREB* gene family.

### 2.2. Phylogenetic Analysis and Structural Prediction of PeAREBs

**Figure 2:**
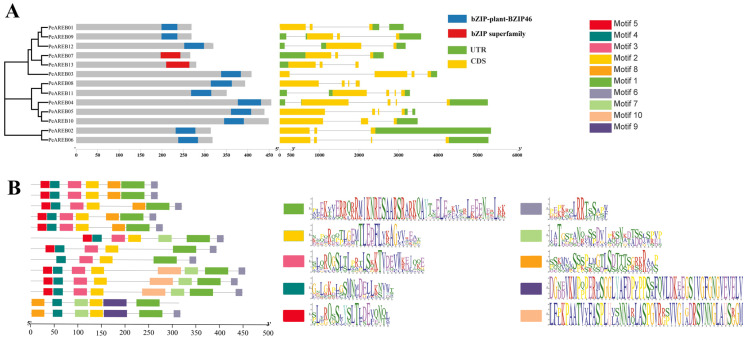
Phylogenetic relationship, gene conserved domain, gene structure and protein conserved domain of PeAREBs conserved protein motifs. (**A**) Neighbor-joining(NJ) phylogenetic tree of 13 PeAREB proteins; conserved domain of *PeAREB* gene family; exon/intron structure of *PeAREBs*. (**B**) Predicting the conserved domain of PeAREBs protein. Different colour boxes represent conserved domains, gene structures and motifs of different gene families.

Analysis of the Pfam conserved structural domains of the *PeAREB* family members revealed that 11 of 13 members contained the bZIP-plant-BZIP46 domain, while *PeAREB07* and *PeAREB13* possessed the bZIP superfamily domain (Figure 2A). According to gene structure annotations, the *PeAREB* gene family exhibited a CDS count ranging from 2 to 4 and a UTR count ranging from 1 to 3.

The MEME analysis of the PeAREBs identified 10 conserved motifs (Figure 2B). The results indicate that PeAREB02 lacks motifs 3, 5 and 7, while PeAREB06 lacks motifs 3 and 5. The remaining PeAREBs contain motifs 1 through 6. Conserved domain prediction using CD Search revealed that all 13 PeAREBs possess highly conserved bZIP (leucine zipper) structures. Additionally, the N-terminal C1 (Motif 5), C2 (Motif 3) and C3 (Motif 2) domains along with the C-terminal C4 (Motif 6) domain contain a conserved phosphorylation motif (RXXS/T), which has been shown to activate AtAREB1 for drought resistance in *Arabidopsis*.

### 2.3. Colinear and Phylogenetic Analysis of AREB Genes Across Multiple Species

**Figure 3:**
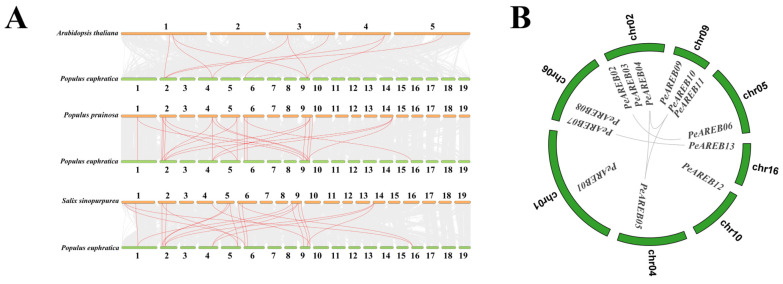
The phylogeny and collinearity of multi-species *AREB* genes, and intraspecific collinearity of *PeAREBs*. (**A**) *P. euphratica*, *P. pruinosa*, *S. purpurea* and *A. thaliana* species collinearity. Numbers represent chromosome numbers. The cyan short bars indicate *P. euphratica* chromosomes, while the yellow short bars at different positions represent *A. thaliana*, *P. pruinosa*, and *S. purpurea*. (**B**) Intraspecific collinearity of *P. euphratica*.

To further investigate the evolutionary history of *AREBs*, we conducted a comparative genomic analysis of *AREB* genes in *P. euphratica* and three other species (*A. thaliana*, *P. pruinosa* and *S. sinopurpurea*, Figure 3A). The results show the following numbers of co-linear *AREB* gene pairs: 9 pairs between *P. euphratica* and *A. thaliana*, 24 pairs with *P. pruinosa* and 22 pairs with *S. sinopurpurea*. Hence, the collinearity of *AREB* genes was more conserved within Salicaceae than between *P. euphratica* and *A. thaliana*. In the *P. euphratica* genome, five co-linear gene pairs (*PeAREB02*/*PeAREB06*, *PeAREB04*/*PeAREB05*, *PeAREB04*/*PeAREB10*, *PeAREB05*/*PeAREB10* and *PeAREB07*/*PeAREB13*) were identified (Figure 3B), suggesting that the evolution of *PeAREB* genes were influenced by segmental duplication events.

**Figure 4:**
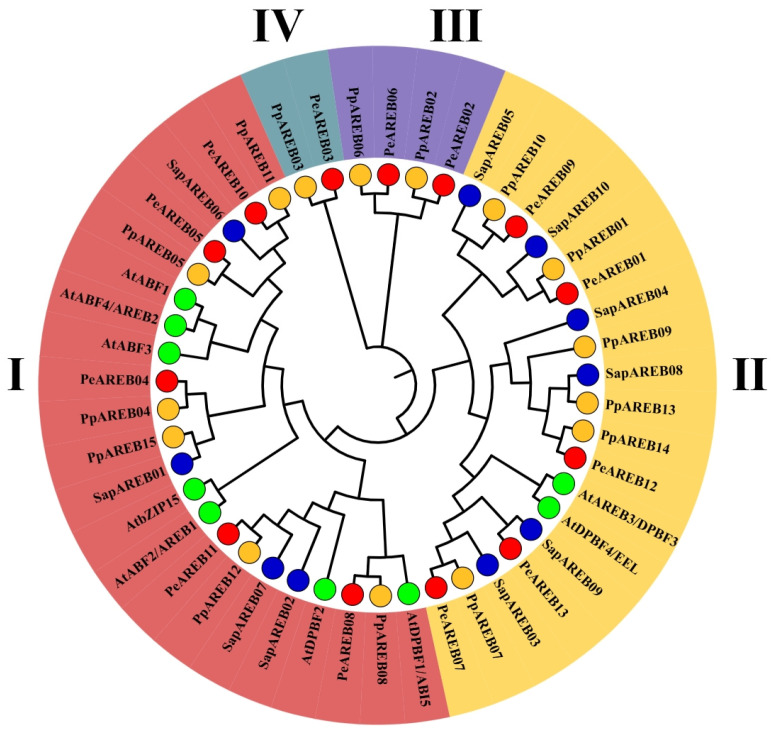
Phylogenetic analysis of AREBs in *P. euphratica*, *P. pruinosa*, *S. purpurea* and *A. thaliana*.

To investigate the evolutionary relationships among AREB gene family proteins in different plant species, we aligned the protein sequences of 13 PeAREBs, 15 PpAREBs, 10 SsAREBs and 9 AtAREBs. A phylogenetic tree was then constructed (Figure 4). Based on the phylogenetic tree, the AREB proteins were categorised into four branches, labeled I to IV. Branch I contained 22 proteins (7 AtAREBs, 5 PeAREBs, 6 PpAREBs and 4 SapAREBs), Branch II contained 19 proteins (2 AtAREBs, 5 PeAREBs, 6 PpAREBs and 6 SapAREBs), Branch III contained 4 proteins (2 PeAREBs and 2 PpAREBs) and Branch IV contained 2 proteins (PeAREB03 and PpAREB03). The presence of all four species in Branches I and II suggests their potential functional similarity. Branches III and IV are unique to Salicaceae. Branches III and IV, containing only *Populus* species, suggested that these genes may play unique roles in adaptive evolution and stress responses specific to *P. euphratica* and *P. pruinosa*. The phylogenetic analysis, along with Figure S1, showed PeAREB04 and AtAREB1 as direct homologous proteins and previous studies reported that AtAREB1 is positively regulated under drought stress in plants.

### 2.4. Prediction of Cis-Acting Elements in the Promoter Regions of PeAREBs

**Figure 5:**
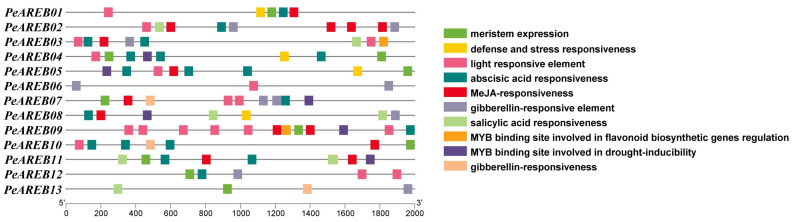
Predicted cis-acting elements in the promoter regions of *PeAREB* gene family members.

The promoter sequences located 2000 bp upstream of the transcription start site of *PeAREB* genes were analysed for cis-acting elements (Figure 5). The promoter regions of *PeAREB* genes contain multiple cis-acting elements involved in responses to stress, hormone signalling, plant growth and development and light response. Additionally, nearly all *PeAREB* gene-promoter regions contained multiple elements associated with responses to abiotic stress. Hormone-related cis-acting elements are dominant in the promoter of *PeAREBs*, such as the ABA-related element *ABRE*. With the exception of *PeAREB06* and *PeAREB13*, the promoters of all other *PeAREB* genes contained MBS elements related to drought response. These findings suggest that *PeAREB* genes may play significant roles in drought stress response and ABA-signalling pathways.

### 2.5. Transcriptional Expression Profile of PeAREB Genes Under Drought Stress

**Figure 6:**
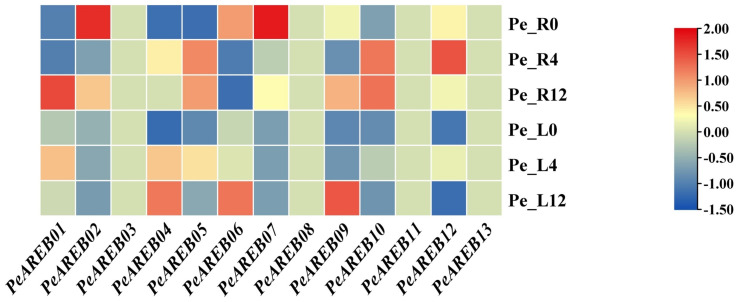
Expression patterns of *PeAREB* genes.

To investigate the response mechanism of *PeAREB* genes to drought stress, a heat map analysis of transcriptome expression profiles was conducted for *P. euphratica* seedling leaves and roots at 0 h, 4 h and 12 h during drought treatment (Figure 6). The results showed that *PeAREB04*, *PeAREB05* and *PeAREB10* were upregulated in both roots and leaves from 0 h to 12 h. Notably, *PeAREB04*, a direct homolog of *AtAREB1* in *A. thaliana*, which is known to play a key role in drought stress response, exhibited the most significant upregulation, with a twofold increase in both roots and leaves. These findings suggest that *PeAREB04* may play an important regulatory role in drought stress response during the seedling stage.

### 2.6. Subcellular Localisation of PeAREB04

**Figure 7:**
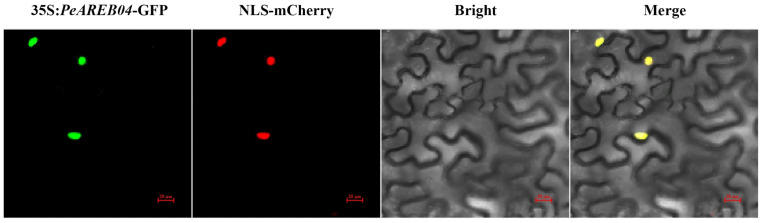
Nuclear localization of the 35S::*PeAREB04*-GFP protein in tobacco leaf epidermal cells. The fluorescence images of PeAREB04 (35S::*PeAREB04*-GFP), nuclear localization signal (NLS-mCherry) and merged image (35S::*PeAREB04*-GFP/NLS-mCherry) were sequentially displayed from left to right. (Scale bar = 20 μm).

To examine the subcellular localisation of the PeAREB04 protein in plant cells, *Agrobacterium tumefaciens* strain GV3101 carrying the 35S::*PeAREB04*-GFP construct, was introduced into tobacco (*Nicotiana benthamiana*). Subcellular localisation of PeAREB04 was then observed using confocal laser scanning microscopy. The results showed that the fluorescence signal of 35S::*PeAREB04*-GFP co-localised with the nuclear localisation signal of NLS-mCherry (Figure 7), indicating that PeAREB04 functions as a nuclear-localised transcription factor.

### 2.7. Overexpression of PeAREB04 Enhances Drought Tolerance in Plants

**Figure 8:**
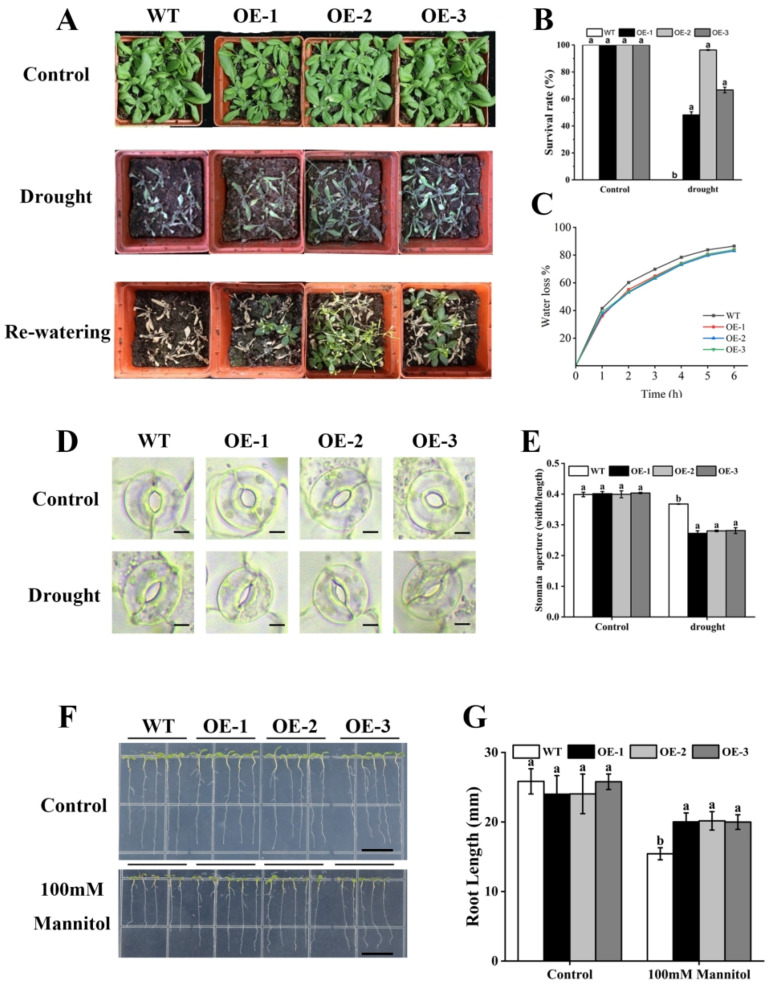
Drought tolerance assays for *PeAREB04* overexpression lines (T3). (**A**) Phenotypes observed in the drought tolerance assays. (**B**) Survival rates of WT and OE lines. (**C**) Water loss rates of detached leaves from WT and OE lines. (**D**) Microscope images showing stomatal openings in WT and three overexpression lines (OE-1, OE-2 and OE-3) under control and drought stress conditions. Scale bar = 5 μm. (**E**) Stomatal opening measurements in leaves of WT, OE-1, OE-2 and OE-3 under control and drought stress conditions. Each treatment was randomly photographed to select 30 stomata. (**F**) Phenotype of WT and OE lines under mannitol treatment. Scale bar = 1 cm. (**G**) Root length measurements of *Arabidopsis* seedlings under control and mannitol treatment conditions. Different letters indicate significant differences based on Duncan’s least significant range test (*p* < 0.05).

The impact of *PeAREB04* overexpression on drought tolerance in 2-week-old *A. thaliana* plants was investigated using 2-week-old *A. thaliana* plants—including overexpression lines OE-1, OE-2 and OE-3 as well as the Col-0 (WT)—under normal conditions and then drought treatment. Compared with the control, the relative expression of *PeAREB04* overexpression lines was significantly up-regulated under drought stress (Figure S2). The OE lines displayed improved growth compared with WT plants under drought conditions (Figure 8A). Following rehydration and an additional 3-day growth period, plant survival was assessed. The OE lines exhibited significantly higher survival rates than WT plants (Figure 8B). The rate of water loss from detached leaves is a critical indicator of drought tolerance in plants. Water loss rates in overexpression lines and WT plants were measured hourly at room temperature. The OE lines exhibited a lower water loss rate than WT plants (Figure 8C).

Stomatal phenotypes of Col-0 and *PeAREB04* overexpression lines (OE-1, OE-2 and OE-3) were analysed using mature rosette leaves from 2-week-old plants. In the light-induced stomatal opening assay, stomatal opening was not significantly different between the WT and OE lines under normal conditions. However, under drought stress, the stomatal openings in the three overexpression lines were significantly smaller than those in the WT (Figure 8D,E). These results indicate that *PeAREB04* negatively regulates stomatal opening under drought conditions. In summary, *PeAREB04* enhanced drought resistance by inhibiting light-induced stomatal opening and reducing transpiration under drought stress. No significant difference in stomatal density was found between WT and overexpression lines (Figure S3). Additionally, the root length of the OE lines was significantly longer than that of WT plants under simulated drought conditions (Figure 8F,G). These results suggest that *PeAREB04* positively regulates plant responses to drought stress.

## 3. Discussion

*P. euphratica* exhibits a strong capacity for adaptation to extreme desert climates; however, drought remains a significant climatic factor that limits the growth of its seedlings. Therefore, investigating the mechanisms of drought resistance in *P. euphratica* is essential to improve seedling survival and stress response [23,24,25]. Under stress conditions, AREB functions as a transcription factor that can specifically bind to the ABA-responsive cis-acting element ABRE, thereby initiating the expression of downstream genes and activating ABA-regulated gene expression [26]. By increasing ABA levels, *ABRE* modulates stomatal opening and influences the expression of related genes to enhance plant resistance to drought stress. *AREB* plays an important role in the drought response of *Arabidopsis* [27,28].

However, the *PeAREB* gene family has not yet been studied. Therefore, we identified the *PeAREB* gene family, found 13 *PeAREB* genes and analysed their response to drought stress in different tissues of *P. euphratica* seedlings. Secondly, the phenotypic changes of overexpressed *Arabidopsis* under drought stress were observed to explore its potential role in plants. Additionally, we identified the *AREB* gene family in *P. pruinosa* and *S. purpurea* to establish a foundation for studying the AREB family in Salicaceae. A total of 47 AREB family members were identified across three Salicaceae species, and *A. thaliana* phylogenetic analyses classified these *AREB* genes into four branches, thereby confirming their evolutionary relationships. The highly conserved structural domains within each branch suggest potential functional similarities. The *AtAREB1* gene has been shown to positively regulate drought stress responses in *A. thaliana*. In this study, we demonstrated that *PeAREB04* is a direct homolog of *AtAREB1*, contains the drought-related cis-acting element MBS and exhibits notable structural and physicochemical similarities. Given that the overexpression of *AtAREB1* enhances drought tolerance in *A. thaliana*, we hypothesised that *PeAREB04* may play a positive regulatory role in drought stress response in *P. euphratica*. This hypothesis warrants further functional validation.

### 3.1. Impact of Genome-Wide Duplication on the Expansion of PeAREBs in the Salicaceae Genus

The origin of multigene families is generally attributed to region-specific or genome-wide duplications, such as polyploidy, which facilitate the establishment of new gene functions and enhance plant adaptation to various environmental stresses [29]. This study revealed that 13 *P. euphratica AREBs* were unevenly distributed across eight chromosomes. The analysis of gene duplication patterns indicated three modes of replication within *PeAREBs*: whole-genome duplication (seven genes), dispersed duplication (five genes) and proximal duplication (one gene), with whole-genome duplication being the primary mode of *PeAREB* amplification. Comparative analysis of *AREB* genes across species demonstrated the highest collinearity between *P. euphratica* and *P. pruinosa* and the lowest with *A. thaliana*. Notably, 7 *AREB* genes were identified on five chromosomes in *Solanum tuberosum* [30,31], 10 *AREB* genes on eight chromosomes in *Oryza sativa* [32] and 14 *AREB* genes on nine chromosomes in *Populus trichocarpa* [33]. This finding suggests a correlation between chromosome number and *AREB* gene count, which could be driven by whole-genome duplication events. Furthermore, AREB proteins in *A. thaliana* localise to the nucleus. In summary, *P. euphratica* contains fewer *AREBs* than *P. pruinosa*, potentially due to gene loss during its adaptation process [34]. The higher number of *AREB* genes in the *P. euphratica* genus compared with *A. thaliana* may reflect adaptations to arid desert environments.

### 3.2. PeAREBs Are Involved in the Regulation of Biological Processes

The role of AREBs in stress response, growth and development has been extensively studied and characterised in *A. thaliana* and *S. tuberosum* [35,36]. AREBs can bind to ABRE cis-acting elements and activate ABA-dependent gene expression under drought stress, demonstrating sensitivity to ABA signalling [37]. AREB transcription factors play crucial roles in secondary metabolism, responses to various stresses and hormonal regulation in higher plants [38]. Members of the *AREB* gene family belong to the A subfamily of bZIP, which is primarily associated with hormonal responses and abiotic stress tolerance. To investigate the functions of *AREB* genes, we analysed their conserved structural domains and found that all *AREB* genes contain C1, C2, C3 and C4 conserved domains, along with regions harbouring potential phosphorylation sites [39]. In *AtAREB1*, these phosphorylation sites are critical to regulating activation sites [40]. Furthermore, all *AREB* genes possess a terminal bZIP structure, suggesting functional roles related to bZIP genes.

To investigate the relationship between *AREB* genes in *P. euphratica* and *A. thaliana*, we constructed a phylogenetic tree by using the NJ method. These proteins were categorised into four distinct branches. The phylogenetic analysis revealed that PeAREBs and AtAREBs clustered together in two major branches, suggesting that *AREB* genes are functionally conserved across species. However, the presence of poplar-specific branches (e.g., PeAREB02, PeAREB03, PeAREB06, PpAREB02, PpAREB03 and PpAREB06) indicates that *AREB* genes may have developed an evolutionary pattern unique to the *Populus* genus following its divergence from *A. thaliana*. *AtAREB1* needs to activate ABRE related to drought stress through ABA for signal transduction. Based on the phylogenetic relationship and Figure S1, *PeAREB04* is a direct homolog of *AtAREB1*. Given that *AtAREB1* has been implicated in abiotic stress response in previous studies, we hypothesised that *PeAREB04* may function in enhancing drought tolerance.

Transcriptome data analysis showed that the expression of *PeAREB04* in the roots and leaves of *P. euphratica* seedlings was significantly upregulated under drought stress, indicating that this gene responded to drought stress. The cis-acting element analysis showed that these genes may regulate gene transcription by binding active transcription factors and cis-acting elements, thereby enhancing plant drought resistance. Functional verification of *AtAREB* genes revealed that ABA and drought stress can activate various *AtAREB* genes [41].

### 3.3. PeAREB04 Enhances Drought Tolerance in Plants

ABA is a crucial plant hormone, with members of the AREB transcription factor family serving as key regulators of ABA-dependent gene expression and playing significant roles in plant hormone signalling and responses to abiotic stress [42,43]. Subcellular localisation analysis indicated that PeAREB04 functions within the nucleus. To investigate the biological function of *PeAREB04*, we subjected the overexpression lines *PeAREB04* to drought stress. The results showed a significantly higher survival rate in overexpression lines compared with WT, along with a notably reduced rate of leaf water loss. Under mannitol-induced stress, the root length of the overexpression lines was markedly greater than that of WT [44]. Under drought stress, the overexpression lines exhibited significantly reduced stomatal aperture relative to WT. These findings suggest that *PeAREB04* enhances plant resistance to drought stress and improves survival rates [45,46]. This study provides a foundation for the functional analysis of the *PeAREB04* gene and supports further exploration of the specific role and drought resistance mechanisms of *PeAREBs* under drought stress.

## 4. Materials and Methods

### 4.1. Identification of PeAREB Gene Family Members and Analysis of Chromosomal Localisation

The *PeAREB* gene family was analysed based on the three-generation genome sequencing data of *P. euphratica* [47]. Hidden Markov Model (HMM) profiles (version 3.0, http://hmmer.org/, accessed on 9 June 2024) corresponding to the bZIP domain and the C1, C2, C3 and C4 structural domains were downloaded from the Pfam (PF00170) protein family database (http://pfam.xfam.org/, accessed on 9 June 2024). These profiles were scanned to identify AREB proteins. Redundant candidate genes were excluded, and SMART was used for additional validation of the remaining genes (http://smart.emblheidelberg.de/, accessed on 11 June 2024). The *AREB* gene was identified based on genomic data from *Populus pruinosa* [48] and *Salix sinopurpurea* [49]. The chromosomal localisation of the *AREB* genes was obtained from the genome annotation files, and the chromosome physical localization of the genes was visualised using MapChart software (version 2.32).

### 4.2. Analysis of Replication Types and Physicochemical Properties of the PeAREB Gene Family

MCScanX software (https://megasoftware.net/, accessed on 20 June 2024) was utilised to identify gene duplication types across the entire *P. euphratica* genome. Duplication patterns specific to *PeAREB* genes were analysed. All duplication types among the *PeAREB* gene family members were quantified based on the GFF3 annotation file and subsequently plotted to illustrate their proportions.

The properties of the identified PeAREB family proteins were analysed by parsing the gene structure annotation files of the known *P. euphratica* genome within our research group. This analysis was conducted using the online tool ExPASy and included physicochemical parameters such as number of amino acid residues, relative molecular weight, aliphatic index, total average hydrophobicity and isoelectric point [50].

### 4.3. Prediction of Conserved Structural Domains, Gene Structure and Motifs of PeAREB Gene Family Members

The GFF3 annotated file for *P. euphratica* was visualised, and the Pfam conserved structural domains along with the gene structures of *P. euphratica* were analysed using TBtools software. The distributions of introns and exons were predicted. The conserved motifs of the PeAREB family were identified using the MEME online tool (https://meme-suite.org/meme/tools/meme, accessed on 15 October 2024), with the number of output motifs set to 10 while retaining the default values for the remaining parameters. Finally, TBtools (version 2.119) was employed to visualise the prediction results [51].

### 4.4. Phylogenetic Analysis of the PeAREB Gene Family

The full-length amino acid sequences of the identified *PeAREB*, *PpAREB*, *SapAREB* and *AtAREB* gene families were compared and analysed to construct a phylogenetic evolutionary tree. The merged protein sequences were compared using MEGA (http://www.example.com). Following the comparison of amino acid sequences, gaps were trimmed using the Multiple Comparison Trimming Tool in TBtools software, with a site coverage cutoff parameter set to 0.95. The NJ method was then employed, and 1000 bootstrap replicates were conducted using MEGA (version 11.0.13). Next to the branch is shown the percentage of related taxa aggregated in the bootstrap test. Dayhoff matrix-based method was utilised to calculate the evolutionary distance, which represents the number of amino acid substitutions per locus. The pairwise deletion option was applied to exclude double-ended positions in each pair of sequences. Phylogenetic trees were visualised using TBtools (version 2.119) and the iTOL online platform (https://itol.embl.de/, accessed on 20 October 2024).

### 4.5. Collinearity Analysis of Multi-Species AREB

TBtools (version 2.119) was utilised to extract chromosome length information (Fasta Stats), *AREB* gene IDs and positional data (GFF3 gene position parsing/Text Block Extraction and Filtering) from the genome files of *P. euphratica*, *P. pruinosa*, *S. sinopurpurea* and *A. thaliana*. The chromosome positions were subsequently visualised using the Gene Location Visualisation function in TBtools (version 2.119) [52,53].

### 4.6. Analysis of Cis-Acting Elements of PeAREBs

The promoter sequences located 2000 bp upstream of the transcription start site of the *PeAREB* genes were extracted using TBtools (version 2.119) based on the GFF3 annotation file. These extracted sequences were then submitted to PlantCare (https://bioinformatics.psb.ugent.be/webtools/plantcare/html, accessed on 20 September 2024) for cis-acting element prediction. Finally, TBtools (version 2.119) was utilised for visualisation and further analysis.

### 4.7. Cloning of the PeAREB04 Gene and Generation of Overexpression Plants

*Escherichia coli* (DH5α), *Agrobacterium rhizogenes* (GV3101) and an overexpression vector (pBWA(V)HS) were maintained in our laboratory. RNA was extracted using Trizol (Invitrogen, Co., Ltd., Carlsbad, CA, USA) and reverse transcribed with the M5 Sprint qPCR RT kit containing gDNA remover (Mei 5 Biotechnology, Co., Ltd., Beijing, China). Phanta Max Super-Fidelity DNA polymerase (Vazyme, Co., Ltd., Nanjing, China) was employed to ensure high fidelity. The full-length coding region of the *PeAREB04* gene, excluding the stop codon, was amplified from cDNA or plasmid with high fidelity. The product was then ligated into pBWA(V)HS by using T4 DNA ligase (Sangon Biotech, Co., Ltd., Shanghai, China). The transformation was carried out in *A. thaliana* through the inflorescence dip method [54,55]. Overexpression lines of *PeAREB04* were established in a WT background, and three lines were selected from the overexpression lines for subsequent functional validation.

### 4.8. Subcellular Localisation of PeAREB04

The constructed 35S::*PeAREB04*-GFP vector was introduced into *A. rhizogenes* GV3101. The transformed bacteria were mixed with NLS-mCherry in equal proportions and inoculated into tobacco leaves by using a syringe. The lower epidermis of tobacco was observed using a laser scanning confocal microscope (Nikon eclipse Ti2, Tokyo, Japan) in a dark environment. A 488 nm laser was used for excitation, and the emission signal was detected within the 500–550 nm range.

### 4.9. Transcriptomic Expression Analysis of PeAREBs

Transcriptome data were obtained from prior publications by our group. Seedlings of *P. euphratica* were treated with a 25% PEG 6000 solution. Leaves and roots were collected at 0, 4 and 12 h, immediately frozen in liquid nitrogen, and sequenced using Ion Proton sequencing at the Beijing Genomics Institute (BGI, Shenzhen, China) [56]. Gene expression was calculated using the RPKM (reads per kilobase of transcript per million mapped reads) method. The RNA-seq data were uploaded to NCBI (https://www.ncbi.nlm.nih.gov/bioproject/PRJNA580347/, accessed on 11 September 2024) [56]. TBtools (version 2.119) was used to visualise the transcriptome data, and numerical standardisation and logarithmic transformation were performed. The logarithmic parameter settings were as follows: base = 2.0; LogWith = 1.0.

### 4.10. Plant Growth Conditions and Drought Stress Treatments

*A. thaliana* seeds (Columbia ecotype, Col-0) were sown on 1/2 MS medium, vernalised at 4 °C for 3 days and then transferred to a growth chamber set at 21 °C with a 16 h light/8 h dark photoperiod. After 3 days, the seedlings were transplanted to moist soil and continued to grow in the growth chamber.

The Col-0 and PeAREB04-OE (T3) plants were grown under normal conditions for 2 weeks, after which watering was stopped for about 10 days, to simulate drought treatment, until the leaves of the plants showed obvious wilting. Photographs of the plants were taken after 10 days to document their growth conditions. Photographs were taken to document growth status. Rehydration treatments were applied, and survival rates were assessed by photographing the plants 3 days after rehydration. For water loss rate determination, the seventh, eighth and ninth leaves were excised from plants grown under normal conditions for 2 weeks, and the blade was left on the filter paper at room temperature. The fresh weight of leaves at each time point was measured at 1, 2, 3, 4, 5 and 6 h, and three biological replicates were performed [57,58].

For mannitol-induced drought stress treatment, Col-0 and *PeAREB04*-OE seeds were sown on 1/2 MS medium, vernalised at 4 °C for 3 days and then transferred to a growth chamber for an additional 3 days. Seedlings with uniform root length were subsequently transplanted onto 1/2 MS medium (control) or 1/2 MS medium supplemented with 100 mM mannitol. After 3 days, seedling growth was documented through photography, and root length was measured. Throughout the experiment, the seedlings were grown vertically, and three biological replicates were performed [59,60,61].

### 4.11. Stomatal Phenotypic Analysis of Rosette Leaves

Col-0 and PeAREB04-OE lines had normal watering and drought treatment. The ninth rosette leaves of comparable size were excised at the base from each genotype. The central region of each leaf was sampled to examine stomatal phenotypes through microscopy at room temperature. For the light-induced stomatal opening experiment, the samples were immersed in MES-KCl buffer, placed under shade for 3 h to induce complete stomatal closure and then illuminated for an additional 3 h to promote stomatal opening [62]. Guard cells were observed using an microscope (Nikon MQD4200, Tokyo, Japan). Thirty stomata were randomly imaged per strain, and stomatal length and width were measured using Image (1.44p) software (stomatal opening = width/length).

### 4.12. Statistical Analysis

Data were represented as means ± standard error. For the analysis of variance (ANOVA) test, different letters indicate significant differences at based on Duncan’s least significant range test (*p* < 0.05).

## 5. Conclusions

In this study, we analysed the gene structures and conserved motifs of 13 identified *PeAREBs* and revealed consistent structural domains, conserved motifs and similar gene structures. Prediction of cis-acting elements, along with detailed analysis of the phylogenetic relationships and collinearity among 47 *AREB* gene family members from three Salicaceae species and *A. thaliana*, indicated high conservation across evolutionary processes. This subfamily is hypothesised to play a crucial role in regulating drought stress through the ABA pathway. RNA-Seq data revealed that *PeAREB04* was significantly upregulated under drought stress. Compared with WT, the *PeAREB04* overexpression lines exhibited higher survival rates, reduced water loss and smaller stomatal openings following drought stress. Under mannitol-induced drought stress, these overexpression lines showed significantly greater root growth relative to the WT, suggesting that *PeAREB04* is instrumental in enhancing drought tolerance. This study establishes a foundation for further functional exploration of *AREB* genes in *P. euphratica* and advances our understanding of drought stress mechanisms in *P. euphratica* through a comprehensive analysis of the *PeAREB* gene family.

## Figures and Tables

**Table 1:** Physicochemical properties of the *PeAREB* gene family.

Sequence ID	Gene ID	Number of Amino Acid	Molecular Weight	Theoretical pI	Instability Index	Aliphatic Index	GRAVY
PeuTF01G02200.1	*PeAREB01*	269	30,279.01	8.36	55.89	63.49	−0.801
PeuTF02G00444.1	*PeAREB02*	314	34,540.42	4.94	54.01	67.07	−0.754
PeuTF02G00696.1	*PeAREB03*	409	46,677.38	9.01	59.66	65.06	−0.78
PeuTF02G01055.1	*PeAREB04*	455	49,251.12	9.42	49.37	68.99	−0.668
PeuTF04G01309.1	*PeAREB05*	439	47,535.9	9.61	48.09	70.05	−0.669
PeuTF05G01689.1	*PeAREB06*	318	35,007.38	5.64	60.93	67.45	−0.744
PeuTF06G00233.1	*PeAREB07*	266	29,801.56	5.52	51.63	70.34	−0.822
PeuTF06G00810.1	*PeAREB08*	394	43,160.06	9.09	52.53	62.46	−0.776
PeuTF09G00191.1	*PeAREB09*	269	30,408.27	9.61	49.15	67.81	−0.817
PeuTF09G01018.1	*PeAREB10*	449	49,487.98	10	46.02	72.96	−0.586
PeuTF09G01642.1	*PeAREB11*	351	39,513.62	7.65	56.89	66.92	−0.848
PeuTF10G00079.1	*PeAREB12*	320	36,118.93	6.93	49.86	68.22	−0.767
PeuTF16G00194.1	*PeAREB13*	280	31,370.03	5.83	61.35	66.14	−0.879

## Data Availability

Data is contained within the article and Supplementary Materials.

## Supplementary Materials

The following supporting information can be downloaded at https://www.mdpi.com/article/10.3390/ijms26020518/s1.

## Abbreviations

AREB: ABA Response Element Binding Protein; ABF: AREB Binding Factors; ABA: Abscisic acid; WT: Wild type; GRAVY: Grand average of hydropathy; GFP: Greenfluorescent protein; OE: Over-Expression; CDS: Coding sequences; UTR: Untranslated Regions; PP2C: Protein phosphatase 2C; SnRK2: Sucrose non-fermenting-1-related protein kinase 2.
