# Supplementary material for: Comprehensive Identification of AREB Gene Family in Populus euphratica Oliv. and Functional Analysis of PeAREB04 in Drought Tolerance
Source: Int J Mol Sci. 2025 Jan 9;26(2):518. doi: 10.3390/ijms26020518 (PMC11764895; doi:10.3390/ijms26020518)
Supplement: Supplementary file 1 [file ijms-26-00518-s001.zip › ijms-3399886-supplementary.pdf]

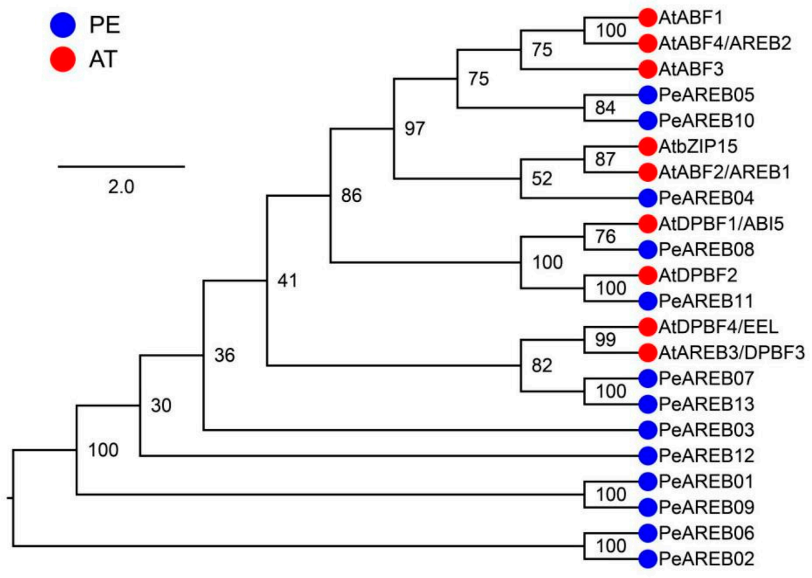

Figure S1. Phylogenetic relationships between *P. euphratica* and *A. thaliana* AREBs

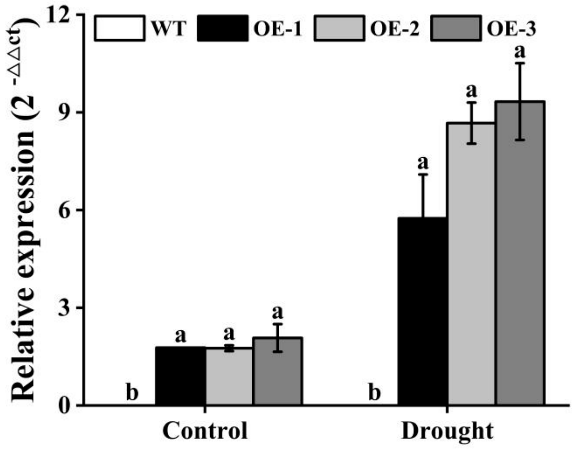

Figure S2. Expression analysis of WT and overexpression line strains

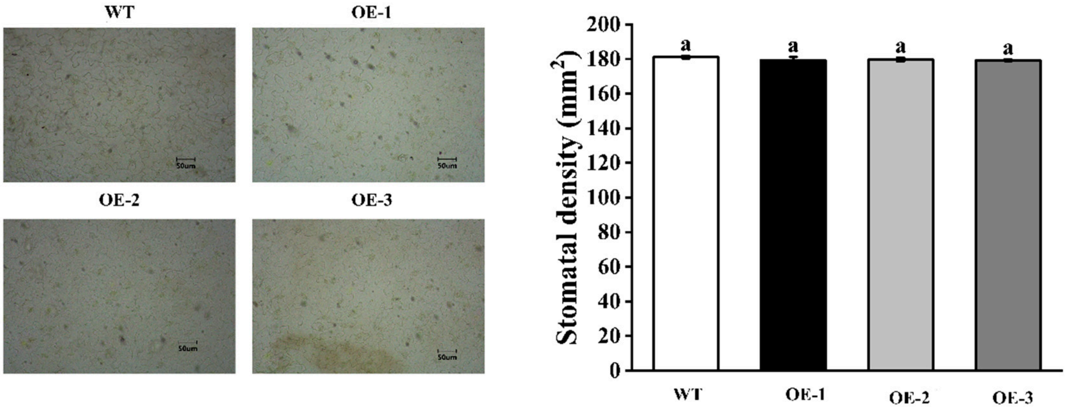

Figure S3. Stomatal density analysis of WT and overexpression line strains
